# Supplementary material for: Improvement of classification performance of Parkinson’s disease using shape features for machine learning on dopamine transporter single photon emission computed tomography
Source: PLoS One. 2020 Jan 24;15(1):e0228289. doi: 10.1371/journal.pone.0228289 (PMC6980558; doi:10.1371/journal.pone.0228289)
Supplement: Attachment — Submitted filename: Response to reviewers20200101#2.docx [file pone.0228289.s002.docx]

We are grateful to reviewers for the critical comments and useful suggestions that have helped us to improve our manuscript considerably. As indicated in the responses that follow, we have taken all these comments and suggestions into account in the revised version of our manuscript.

line 130ff: the authors still do not specify if striatal activity was differentiating caudate activity from putaminal activity. It seems they just differentiated left striatal activity from right striatal activity. They might insert a line "left and right striatal ROIs were covering and including all activity visualised in putamen and caudate"

Response

We strongly appreciate the reviewer’s comment. In accordance with reviewer’s comment, we have changed the text in the Materials and Methods as from:

“Regions of interests (ROIs) were placed on the left and right caudate and putamen (target region) and the occipital cortex (reference region).”

to

“Regions of interests (ROIs) were placed on the left and right striatal ROIs were covering and including all activity visualised in putamen and caudate (target region), and the occipital cortex (reference region).” (P9L133)

Line 113 typo PPIM instead of PPMI

Response

We strongly appreciate the reviewer’s comment. In accordance with reviewer’s comment, we have revised from “PPIM” to “PPMI”(P8L113).

Line 114: "SPECT images and SBRs were downloaded from the PPMI website" - please change to "Preprocessed SPECT images and SBRs were downloaded from the PPMI website".

Response

We strongly appreciate the reviewer’s comment. In accordance with reviewer’s comment, we have changed the text in the Materials and Methods as from:

“SPECT images and SBRs were downloaded from the PPMI website.”

to

“Preprocessed SPECT images and SBRswere downloaded from the PPMI website.” (P8L114)

Line 114 ff: "SPECT imaging was acquired at each imaging centers as per the PPMI imaging protocol..." The current wording suggests that this part of processing had been done by the authors. In fact the whole paragraph following is plagiating the PPMI methods section where the preprocessing steps performed by PPMI are described, which is misleading the reader. Please consider the following wording:
"As by PPMI documentation, preprocessing steps were performed at the Institute for Neurodegenerative Disorders (IND, New Haven, CT) and included the following steps: SPECT imaging and reconstruction: SPECT imaging "was acquired at each imaging centers as per the PPMI imaging protocol [..., up to line 127]. (line 128 insert:) The calculation method of SBR as performed at the IND was as follows: the transaxial slice with...

Response

We strongly appreciate the reviewer’s comment. In accordance with reviewer’s comment, we have added the text in the Materials and Methods as follows(P8L114):

As by PPMI documentation, preprocessing steps were performed at the Institute for Neurodegenerative Disorders (IND, New Haven, CT) and included the following steps: SPECT imaging and reconstruction:

Also, we have changed the text (P9L131) as following:

The calculation method of SBR as performed at the IND was as follows: the transaxial slice with the highest striatal uptake was identified, and the eight hottest striatal slices around it were averaged to generate a single slice image.

line 146: " The region where the radioactivity is visually accumulated at the site where the striatum exists anatomically was surrounded" - please replace "surrounded" by "manually delineated"

Response

We strongly appreciate the reviewer’s comment. In accordance with reviewer’s comment, we have changed the text in the Materials and Methods as from:

“The region where the radioactivity is visually accumulated at the site where the striatum exists anatomically was surrounded.”

to

“The region where the radioactivity is visually accumulated at the site where the striatum exists anatomically was manually delineated.” (P10L151)

Additional comment: reviewer 1 asked for definition of "burst striatum" which is not given in the revised document.4 Please provide.

Response

We strongly appreciate the reviewer’s comment. In accordance with reviewer’s comment, we have added the text in the Materials and Methods (P7L105) as following:

The burst striatum type is severe bilateral reduction with almost no uptake in either the putamen or caudate[7].

We wish to thank the Reviewer again for his or her valuable comments.
